# Promoting postoperative recovery in older adult surgical patients: a randomized controlled trial of MDT-based perioperative comprehensive nutritional management model

**DOI:** 10.3389/fnut.2025.1684807

**Published:** 2026-01-12

**Authors:** Qi An, Yonghao Li, Jinxin Shi, Chengyu Liu, Zijian Li, Liru Chen, Linlin Gao, Lei Li, Qingmei Liu, Hongyuan Cui, Huan Xi, Mingwei Zhu

**Affiliations:** 1 Department of General Surgery, Gastrointestinal Surgery, Beijing Hospital, National Center of Gerontology, Institute of Geriatric Medicine, Chinese Academy of Medical Sciences, Beijing, China; 2 Department of General Surgery, Department of Hepato-Bilio-Pancreatic Surgery, Beijing Hospital, National Center of Gerontology, Institute of Geriatric Medicine, Chinese Academy of Medical Sciences, Beijing, China; 3 Chinese Academy of Medical Sciences, Peking Union Medical College, Beijing, China; 4 Department of General Surgery, Peking Union Medical College Hospital, Chinese Academy of Medical Sciences and Peking Union Medical College, Beijing, China; 5 Department of Clinical Nutrition, Beijing Hospital, National Center of Gerontology, Institute of Geriatric Medicine, Chinese Academy of Medical Sciences, Beijing, China; 6 Department of Rehabilitation Medicine, Beijing Hospital, National Center of Gerontology, Institute of Geriatric Medicine, Chinese Academy of Medical Sciences, Beijing, China

**Keywords:** comprehensive nutritional management, older adult patients, perioperative period, multidisciplinary team, postoperative complications, clinical outcomes

## Abstract

**Background:**

Traditional perioperative care is hospital-centric, whereas the efficacy of multidisciplinary team (MDT)-based comprehensive nutrition management remains debated. This study examines how this model impacts postoperative complications and functional recovery in older adults.

**Methods:**

A single-center superiority randomized controlled trial (RCT; April 2023–March 2024) randomized 120 older adults (≥65y) undergoing major abdominal surgery (grades 3–4) into intervention (*n* = 60) and control (*n* = 60) groups, excluding those with severe organ dysfunction or poor compliance. The MDT-based model included personalized exercise, nutrition, psychological support (3 weekly sessions, smoking/alcohol cessation), and post-discharge monitoring via wearables/WeChat. Control group received standard care.

**Results:**

Among 120 participants (mean age 72.6 years; 65.2% male), 5 were lost to follow-up, leaving 56 in the intervention group and 59 in the control group for analysis. The intervention group demonstrated significantly lower postoperative complications compared to controls, with a relative reduction in in-hospital events and lower post-discharge rates (3.6% vs. 6.8%). The Comprehensive Complication Index averaged 6.2 points lower in the intervention group (*p* = 0.175). Body composition improved significantly, showing reduced weight loss, reduced BMI loss, and increased appendicular skeletal muscle mass index. Postoperative hospital stay was shortened by a median of 3.67 days in the intervention group, which also reported higher rates of mobility improvement and pain relief (all *p* < 0.05).

**Conclusion:**

The MDT-based perioperative model synergistically reduces postoperative complications and accelerates geriatric functional recovery through nutritional, exercise, and psychological interventions, providing evidence-based support for high-risk elderly care.

## 1 Background

Surgical demand is rising globally due to aging populations, with cancer-related surgeries projected to increase by 5 million cases from 2018 to 2040 (1). Despite technical advancements, postoperative complications remain high (19.7–37.4% within 30 days), including infections and cardiopulmonary events (2), leading to prolonged hospital stays, increased costs, and elevated mortality risk (3). Older adults face heightened risks due to reduced physiological reserve, comorbidities, and malnutrition (4). Traditional perioperative care focuses on inpatient management (e.g., minimally invasive techniques, early oral intake), but evidence supports prehabilitation with nutritional interventions to improve resilience and reduce hospital stays (5). However, the efficacy of multidimensional prehabilitation (combining nutrition, exercise, and psychology) in reducing complications remains debated. Perioperative comprehensive nutritional management, spanning preoperative, postoperative, and 30-day post-discharge phases, is recognized as critical for optimizing outcomes (6–9). While MDT models are well-established in oncology, their application in surgical nutritional management is understudied (10). Integrating digital tools (e.g., wearables) could enhance adherence but lacks standardization (11).

This study aims to validate the effectiveness of MDT-based perioperative nutritional management in reducing complications and supporting rehabilitation in older surgical patients, providing evidence for geriatric perioperative care paradigms.

## 2 Methods

### 2.1 Study design and patient selection

This study was conducted as an interventional randomized controlled clinical trial in the General Surgery Department of Beijing Hospital between April 1, 2023, and March 31, 2024. A total of 526 patients were screened, and data analysis was performed from September 1 to December 31, 2024. In accordance with the Declaration of Helsinki, the trial protocol was approved by the Ethics Committee of Beijing Hospital (Ethics Approval Number: 2021BJYYEC-305-02) and registered on www.chictr.org.cn (Registration Number: ChiCTR2100054908). All participating patients provided written informed consent. This study adhered to the Consolidated Standards of Reporting Trials (CONSORT) guidelines.

**Figure 1:**
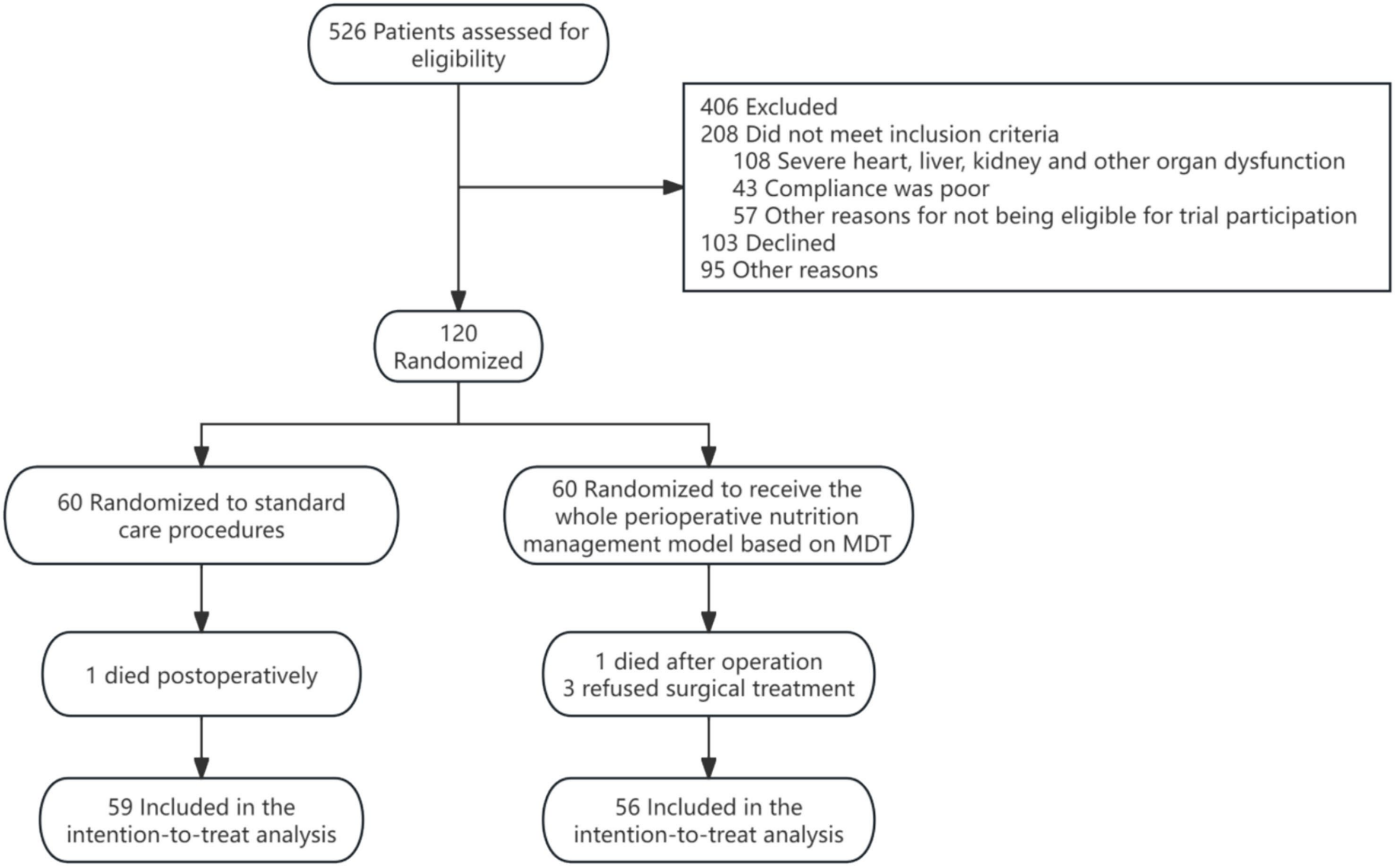
Flowchart of patient screening in this study.

Inclusion Criteria: ① Older adult patients aged ≥65 years; ② Scheduled to undergo major abdominal surgery for diseases of the stomach, colorectum, liver, gallbladder, or pancreas; ③ Baseline liver and kidney function within normal limits (key indicators ≤2 times the upper limit of normal); ④ Ability to tolerate oral intake and ambulate independently preoperatively; ⑤ Provision of written informed consent. Exclusion Criteria: ① Severe gastrointestinal dysfunction precluding oral intake; ② Severe liver, kidney, or other organ dysfunction; ③ Poor treatment compliance; ④ Allergy to nutritional supplements; ⑤ Concurrent participation in other clinical trials or other exclusionary conditions. Figure 1 illustrates the patient screening flowchart for this study.

### 2.2 Study design and sample size estimation

This RCT employed a 1:1 randomization design using IBM SPSS 27.0-generated codes, assigning eligible participants to intervention or control groups after baseline eligibility confirmation. While investigators and participants were unblinded, follow-up assessors and statistical analysts remained blinded to group allocation. Sample size was calculated via PASS 15 based on prior geriatric complication rates (15% intervention vs. 38% control), with α = 5%, power = 80%, yielding 54 participants per group. After adjusting for 10% attrition, the final enrollment target was 120 (60/group).

### 2.3 Intervention of perioperative nutrition management based on MDT

The intervention group received MDT-guided comprehensive perioperative nutritional management adhering to domestic guidelines, involving surgeons, nutritionists, rehabilitation specialists, pharmacists, and nurses who conducted regular consultations. Protocols spanned preoperative, postoperative, and 30-day post-discharge phases, following a structured workflow of screening, assessment, diagnosis, intervention, and monitoring. Key interventions included: ① Tailored nutritional support (ONS/EN/PN for at-risk patients), ② Individualized exercise plans (aerobic: brisk walking ≥5×/week, 30 + mins/session; resistance: squats, 10 reps/set, 2×/day), and ③ Psychological support via relaxation techniques, audio/video resources, and lifestyle counseling. Nutritionists used WeChat for dietary guidance, while smart bracelets tracked heart rate/activity to enhance post-discharge compliance. Smart bracelets were used to monitor heart rate and step count, and WeChat was employed for weekly reminders and dietary guidance to enhance post-discharge compliance. It is worth noting that this does not include real-time AI-driven adaptation.

The intervention group followed a three-phase protocol: Preoperative multidimensional prehabilitation (ONS 500 kcal/d, exercise, counseling) lasted ≥7 days (NRS 2002 3–5) or ≥10 days (NRS > 5). Postoperative care prioritized early oral intake; tube feeding/PN was initiated if oral energy remained <60% of target (25 kcal/kg/d) by 72 h or contraindicated. Post-discharge (30 days) continued ONS, exercise, and weekly WeChat/telephone reminders. The control group received routine care with postoperative nutrition support triggered by similar criteria (oral intake <60% target by 72 h). Specifically, during the preoperative stage, patients receive routine standardized guidance from surgeons and nutritionists, mainly including: ① Arrangement of preoperative fasting time; ② Dietary assessment upon admission to confirm whether the nutritional intake is adequate, etc. Postoperatively, patients are encouraged to engage in early activities, and they follow the guidance of surgeons and nutritionists to resume their diet. After discharge, patients receive routine dietary and exercise guidance and education. Both groups required clinical stability, semi-liquid diet tolerance, and IV independence for discharge.

### 2.4 Outcome measures

The primary outcome was postoperative complications (in-hospital and 30-day post-discharge), defined as any deviation from the ideal postoperative course (e.g., infections, anastomotic leaks, anemia, electrolyte disturbances, myocardial infarction), excluding untreated primary disease. Complications were graded using the Clavien-Dindo system and quantified via the Comprehensive Complication Index (CCI, range 0–100), calculated using an online tool^1^ (12, 13). Patients with fatal outcomes (CCI = 100) were excluded from follow-up.

Secondary outcomes included: BMI; NRS-2002 and Fried frailty scores; body composition metrics (muscle mass, skeletal muscle mass, fat distribution); laboratory parameters (CRP, lipids, albumin, prealbumin, liver/kidney function); clinical metrics (total/postoperative hospital stay, hospitalization costs, 30-day unplanned readmission rates excluding chemotherapy-related admissions).

Additional assessments comprised: EQ-5D quality-of-life questionnaire (mobility, self-care, pain, anxiety, health status); disease-specific evaluations including Fried frailty scale, sarcopenia diagnosis (ASMI, handgrip strength, gait speed), and malnutrition screening (NRS-2002)/diagnosis (GLIM criteria). Follow-up occurred at three timepoints: admission, discharge, and 30 days post-discharge.

### 2.5 Data analysis

All statistical analyses adhered to the modified intention-to-treat principle. An electronic data capture and management system was implemented with double data entry by two independent operators, ensuring raw data preservation and traceable authenticity through dedicated verification and cleaning protocols. Statistical analyses were performed using IBM SPSS Statistics (Version 27.0.1) and R software (Version 4.4.2). Continuous variables are presented as mean ± standard deviation (SD) or median (interquartile range, IQR), while categorical variables are described as frequencies (percentages). Between-group comparisons employed appropriate statistical methods based on data distribution: independent samples t-tests or one-way ANOVA for normally distributed continuous variables, non-parametric tests (Mann–Whitney U/Kruskal-Wallis H tests) for non-normal distributions, and χ^2^ tests or Fisher’s exact tests for categorical variables. Repeated measures analysis within general linear models was used to evaluate significant between-group differences in longitudinal clinical outcomes (baseline, discharge, and 30-day post-discharge), with trend visualization through line graphs. All tests were two-tailed, and statistical significance was defined as *p* < 0.05.

## 3 Results

### 3.1 Baseline patient data

The study screened 526 patients, ultimately enrolling 120 eligible older adults undergoing abdominal surgery. Five participants were lost to follow-up: 2 postoperative deaths (intervention group on day 7, control group on day 5) and 3 withdrawals due to refusal of surgery. The final analysis included 56 patients in the intervention group and 59 in the control group (Table 1). Baseline characteristics were well-matched between groups (all *p* > 0.05), with no significant differences in age (72.36 ± 6.64 vs. 72.80 ± 6.15 years), gender (66.1% vs. 64.4% male), gastrointestinal surgery proportion (58.9% vs. 57.6%), nutritional markers (albumin 37.20 ± 3.37 vs. 37.12 ± 4.68 g/L; prealbumin 19.56 ± 5.55 vs. 20.32 ± 6.11 g/L), or inflammatory/metabolic parameters. Nutritional risk (NRS-2002 ≥ 3: 85.7% vs. 83.1%) and prevalence of prefrailty/frailty (71.4% vs. 71.2%) or sarcopenia (12.5% vs. 18.6%) were balanced at baseline. These comparable baseline features establish a solid foundation for subsequent efficacy evaluation while minimizing confounding factors.

**Table 1:** Baseline demographics, clinical characteristics, and nutritional status of participants in the intervention and control groups.

Clinical features	All patients (*n* = 115)	Intervention groups (*n* = 56)	Control groups (*n* = 59)	*p*
Sex				0.851
Male	75 (65.2%)	37 (66.1%)	38 (64.4%)	
Female	40 (34.8%)	19 (33.9%)	21 (35.6%)	
Age (year)				0.710
	72.58 (2.87)	72.36 (6.64)	72.80 (6.15)	
Marital status				0.487
Married	114 (99.1%)	55 (98.2%)	59 (100.0%)	
Others (divorced, widowed, single, etc.)	1 (0.9%)	1 (1.79%)	0 (0.0%)	
Ethnicity				0.970
Han Chinese	113 (98.3%)	55 (98.2%)	58 (98.3%)	
Others	2 (1.7%)	1 (1.8%)	1 (1.7%)	
Weight (kg)				0.398
	64.82 (10.26)	65.64 (9.56)	64.01 (10.92)	
BMI				0.133
	23.78 (2.87)	24.19 (3.05)	23.38 (2.64)	
Upper arm circumference (cm)				0.103
	27.03 (2.62)	27.35 (2.62)	26.72 (2.61)	
Handgrip strength (kg)				0.356
	27.85 (8.06)	28.65 (8.45)	27.10 (7.69)	
Calf circumference (cm)				0.237
	34.34 (3.43)	34.65 (8.45)	34.04 (3.33)	
Body water (%)				0.875
	34.27 (5.71)	34.42 (5.46)	34.13 (5.81)	
Muscle mass (kg)				0.276
	41.07 (9.37)	42.15 (9.75)	40.05 (9.24)	
Fat-free mass (kg)				0.932
	46.34 (7.58)	46.41 (7.33)	46.28 (7.89)	
Skeletal muscle mass (kg)				0.849
	24.61 (4.64)	24.77 (4.83)	24.46 (4.55)	
Body fat mass (kg)				0.113
	17.52 (6.37)	18.54 (6.79)	16.55 (5.67)	
Body fat percentage (%)				0.403
	27.99 (7.68)	28.70 (8.35)	27.32 (6.65)	
Appendicular skeletal muscle mass (kg)				0.582
	19.28 (4.02)	19.44 (3.92)	19.13 (4.13)	
Appendicular skeletal muscle index (kg/m^2^)				0.461
	6.98 (1.19)	6.92 (1.40)	7.04 (1.17)	
Fat-free mass index (kg/m^2^)				0.801
	17.00 (1.77)	17.09 (1.76)	16.93 (1.77)	
WBC (×10^9^/L)				0.236
	6.29 (3.05)	5.96 (1.84)	6.63 (3.91)	
CRP (mg/L)				0.173
	6.45 (17.17)	10.77 (22.02)	6.03 (9.57)	
Blood glucose (mmol/L)				0.132
	5.35 (1.07)	5.47 (0.94)	5.87 (1.73)	
Total cholesterol (mmol/L)				0.992
	4.09 (1.01)	4.09 (0.88)	4.09 (1.16)	
Triglycerides (mmol/L)				0.755
	1.27 (0.60)	1.29 (0.53)	1.25 (0.65)	
Creatinine (μmol/L)				0.440
	70.18 (22.66)	71.75 (22.37)	68.55 (23.03)	
Urea (mmol/L)				0.862
	5.23 (2.62)	5.19 (2.29)	5.28 (2.95)	
Total bilirubin (μmol/L)				0.234
	24.74 (41.20)	20.30 (34.56)	29.40 (47.06)	
ALT (U/L)				0.807
	29.53 (63.74)	28.11 (72.78)	30.98 (53.41)	
AST (U/L)				0.654
	33.83 (66.67)	31.13 (69.67)	36.63 (63.89)	
Total protein (g/L)				0.354
	62.81 (6.17)	62.64 (5.17)	62.98 (6.33)	
Albumin (g/L)				0.950
	37.16 (4.05)	37.20 (3.37)	37.12 (4.68)	
Prealbumin (mg/L)				0.545
	19.94 (5.82)	19.56 (5.55)	20.32 (6.11)	
Unintentional weight loss				0.265
Yes	59 (51.3%)	32 (57.1%)	27 (45.8%)	
No	56 (48.7%)	24 (42.9%)	32 (54.2%)	
Slowed walking speed				0.323
Yes	40 (34.8%)	22 (39.3%)	18 (30.5%)	
No	75 (65.2%)	34 (60.7%)	41 (69.5%)	
Weak handgrip strength				0.977
Yes	33 (28.7%)	16 (28.6%)	17 (28.8%)	
No	82 (71.3%)	40 (71.4%)	42 (71.2%)	
Low physical activity level				0.958
Yes	4 (3.5%)	2 (3.6%)	2 (3.4%)	
No	111 (96.5%)	54 (96.4%)	57 (96.6%)	
Self-reported exhaustion				0.846
Yes	13 (11.3%)	6 (10.7%)	7 (11.9%)	
No	102 (88.7%)	50 (89.3%)	52 (88.1%)	
Fried frailty score				0.688
	1.23 (1.09)	1.29 (1.17)	1.17 (0.99)	
Frailty status				0.773
Normal	33 (28.7%)	16 (28.6%)	17 (28.8%)	
Pre-frail state	70 (60.9%)	33 (58.9%)	37 (62.7%)	
Frail state	12 (10.4%)	7 (12.5%)	5 (8.5%)	
Decreased appendicular skeletal muscle index				0.791
Yes	36 (31.3%)	15 (37.9%)	21 (35.6%)	
No	79 (68.7%)	41 (62.1%)	38 (64.4%)	
6-meter walk test speed: <1.0 m/s				0.166
Yes	38 (33.0%)	22 (39.3%)	16 (27.1%)	
No	77 (67.0%)	34 (60.7%)	43 (72.9%)	
Sarcopenia status				0.584
Normal	97 (84.3%)	49 (87.5%)	48 (81.4%)	
Yes, non-severe	14 (12.2%)	5 (8.9%)	9 (15.3%)	
Yes, severe	4 (3.5%)	2 (3.6%)	2 (3.4%)	
NRS 2002 total score				0.453
1–2	18 (15.7%)	8 (14.3%)	10 (16.9%)	
3–4	85 (73.9%)	44 (78.6%)	41 (69.5%)	
≥5	12 (10.4%)	4 (7.1%)	8 (13.6%)	
GLIM malnutrition diagnosis				0.913
No	68 (59.1%)	32 (57.1%)	36 (61.0%)	
Moderate	41 (35.7%)	21 (37.5%)	20 (33.9%)	
Severe	6 (5.2%)	3 (5.4%)	3 (5.1%)	
Surgical procedure type				0.970
Gastrointestinal surgery	67 (58.3%)	33 (58.9%)	34 (57.6%)	
Hepatobiliary surgery	32 (27.8%)	15 (26.8%)	17 (28.8%)	
Others	16 (13.9%)	8 (14.3%)	8 (13.6%)	

### 3.2 Comparison of nutritional supplementation duration and intake between groups

In the intervention group, patients received preoperative ONS for a mean duration of 8.27 ± 5.25 days. Postoperatively, 10 patients (17.9%) required tube feeding (EN) for a mean of 9.86 ± 7.13 days, while 34 patients (60.7%) received PN for a mean of 7.12 ± 5.10 days. The average daily total energy intake in the intervention group was 1,558.1 ± 411.6 kcal. In contrast, the control group included 16 patients (27.1%) who received postoperative tube feeding (EN) until discharge, with a mean duration of 11.53 ± 9.32 days, and 49 patients (83.1%) who received PN for a mean of 7.64 ± 6.79 days. The average daily total energy intake in the control group was 1,369.5 ± 319.2 kcal. Notably, 56 patients in the intervention group achieved >80% adherence to prescribed exercise durations and nutritional supplementation targets during the post-discharge intervention period.

### 3.3 Comparison of clinical outcomes between groups

The intervention group demonstrated a significantly lower incidence of postoperative complications compared to the control group (16.1% vs. 32.2%, *p* = 0.044). While in-hospital complication rates showed a non-significant reduction trend (16.1% vs. 30.5%, *p* = 0.068), post-discharge rates were comparable (3.6% vs. 6.8%, *p* = 0.598). We believe that this might be due to the low incidence of individual complications or other interfering factors. Therefore, the statistical significance of the composite outcome reflects the intervention’s effect in reducing the overall incidence of complications, rather than the significance of each individual component. The intervention group achieved a 6.2-point lower CCI score and significantly shorter median postoperative hospital stay. During 30-day follow-up, the intervention group showed greater improvements in mobility (19.6% vs. 3.4%, *p* = 0.018) and pain/discomfort (55.4% vs. 27.1%, *p* = 0.008), with fewer cases of worsening anxiety/depression.

Although total hospital stay (15.81 vs. 18.36 days, *p* = 0.052) and costs (¥68,245.0 vs. ¥77,845.5, *p* = 0.738) did not reach statistical significance, the intervention group exhibited a potential economic benefit with a 2.55-day shorter mean stay and 9.7% cost reduction. Self-reported health status showed a non-significant improvement trend (30.4% vs. 25.4%, *p* = 0.350), while other functional domains (e.g., self-care, daily activities) remained comparable between groups, possibly due to the follow-up duration or assessment tool sensitivity (Table 2).

**Table 2:** Comparison of clinical outcomes between intervention and control groups.

Clinical outcomes	Intervention groups (*n* = 56)	Control groups (*n* = 59)	*p*	X^2^/t
Complications			0.044	4.059
Yes	9 (16.1%)	19 (32.2%)		
No	47 (83.9%)	40 (67.8%)		
Complications (during hospitalization)			0.068	3.333
Yes	9 (16.1%)	18 (30.5%)		
No	47 (83.9%)	41 (69.5%)		
Complications (30 days after discharge)			0.439	0.598
Yes	2 (3.6%)	4 (6.8%)		
No	54 (96.4%)	55 (93.2%)		
Comprehensive complication index			0.175	1.363
	11.9 (23.6)	18.1 (25.1)		
Length of hospital stay			0.052	1.940
	15.81 (4.45)	18.36 (8.77)		
Postoperative length of stay			0.007	2.700
	8.33 (3.15)	12.00 (9.33)		
Hospitalization cost			0.738	0.330
	68245.0 (54504.5,81200.5)	77845.5 (61366.5,100209.3)		
Readmission			1.00	1.343
Yes	0 (0.0%)	1 (1.7%)		
No	56 (100.0%)	58 (98.3%)		
Mobility domain^*^			0.018	8.029
Improvement	11 (19.6%)	2 (3.4%)		
Worsening	3 (5.4%)	6 (10.2%)		
No change	42 (75.0%)	51 (86.4%)		
Self-care domain^*^			0.256	3.305
Improvement	3 (5.4%)	0 (0.0%)		
Worsening	3 (5.4%)	4 (6.8%)		
No change	50 (89.3%)	55 (93.2%)		
Usual activities domain^*^			0.869	0.282
Improvement	6 (10.7%)	5 (8.5%)		
Worsening	8 (14.3%)	10 (16.9%)		
No change	42 (75.0%)	44 (74.6%)		
Pain/discomfort domain^*^			0.008	9.558
Improvement	31 (55.4%)	16 (27.1%)		
Worsening	9 (16.1%)	14 (23.7%)		
No change	16 (28.6%)	29 (49.2%)		
Anxiety/depression domain^*^			0.547	1.208
Improvement	15 (26.8%)	15 (25.4%)		
Worsening	2 (3.6%)	5 (8.5%)		
No change	39 (69.6%)	39 (66.1%)		
Self-rated health^*^			0.839	0.35
Improvement	17 (30.4%)	15 (25.4%)		
Worsening	25 (44.6%)	28 (47.5%)		
No change	14 (25.0%)	16 (27.1%)		
Frailty score change^*^			0.077	5.125
Improvement	15 (26.8%)	11 (18.6%)		
Worsening	3 (5.4%)	11 (18.6%)		
No change	38 (67.9%)	37 (62.7%)		
Sarcopenia progression^*^			0.180	3.689
Improvement	7 (12.5%)	5 (8.5%)		
Worsening	1 (1.8%)	6 (10.2%)		
No change	48 (85.7%)	48 (81.4%)		
Nutritional status change^*^			0.639	1.121
Improvement	13 (23.2%)	11 (18.6%)		
Worsening	3 (5.4%)	6 (10.2%)		
No change	40 (71.4%)	42 (71.2%)		

* 30 days post-discharge vs. at discharge.

### 3.4 Comparison of changes in relevant factors between groups

**Figure 2:**
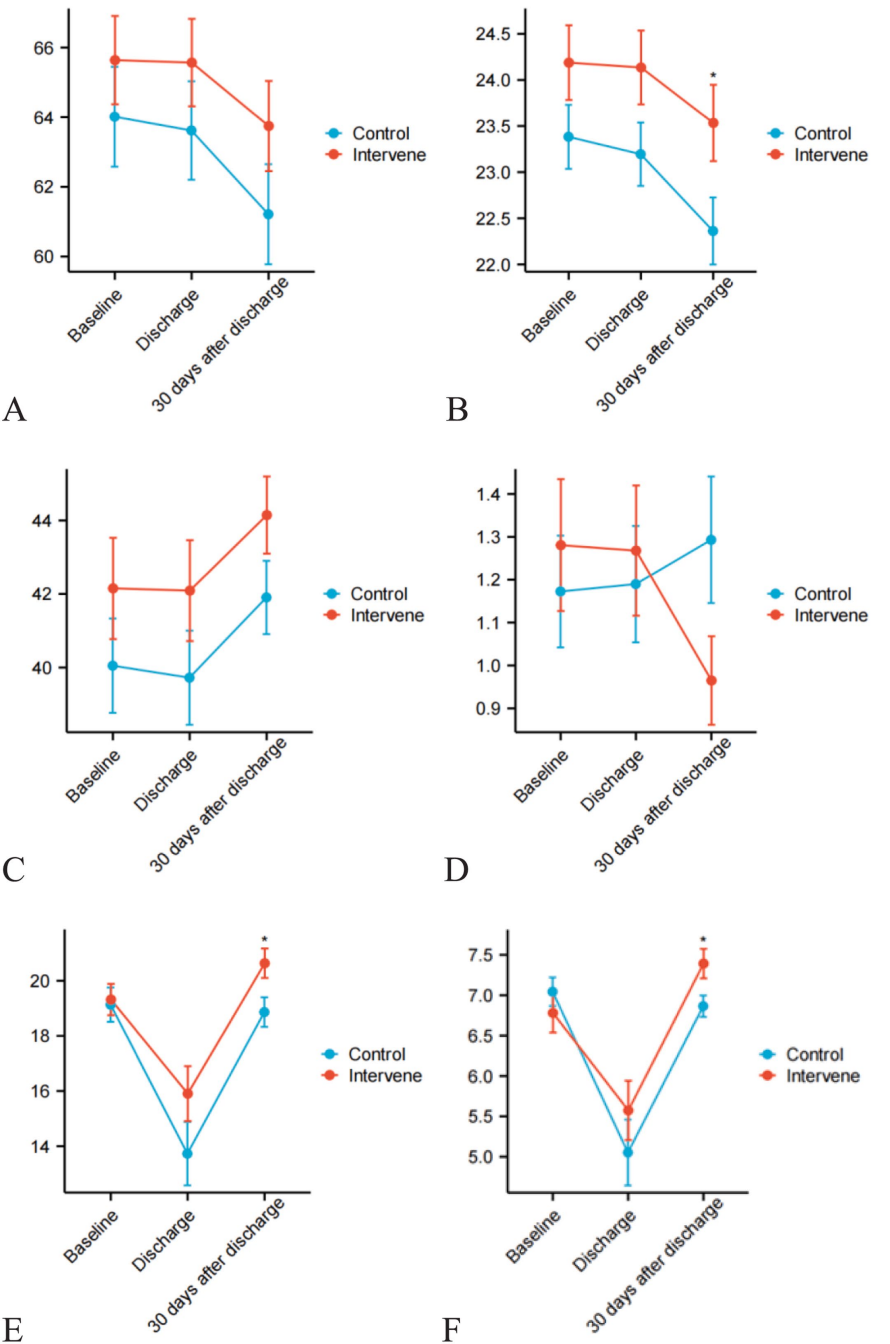
Line graphs depicting temporal changes in clinical parameters between intervention and control groups. **(A)** weight; **(B)** BMI; **(C)** muscle mass; **(D)** frailty index; **(E)** appendicular skeletal muscle mass; **(F)** appendicular skeletal muscle index.

Repeated-measures ANOVA identified significant group-by-time interactions in clinical indicators (Table 3). The intervention group showed superior overall mean levels in total protein, albumin, weight, BMI, grip strength, calf circumference, muscle mass, ASMM, ASMI, and NRS-2002 scores (all *p* < 0.05). Significant time-dependent effects were observed for weight (*p* = 0.040), BMI (*p* = 0.029), ASMM (*p* < 0.001), and Fried frailty scores (*p* = 0.024), indicating enhanced recovery trajectories with MDT nutritional management (Figure 2).

**Table 3:** Longitudinal comparison of clinical parameters at baseline, discharge, and 30-day post-discharge between groups.

Group	Baseline	At discharge	30 Days post-discharge	P_1_	P_2_	Change from Baseline to 30 Days post-discharge (Δ)	P_3_
Total protein (g/L)				<0.001	0.764		0.851
Intervention groups	62.64 (5.17)	57.66 (5.71)	66.27 (8.05)			3.64 (8.04)	
Control groups	62.98 (6.33)	57.12 (6.87)	66.93 (5.40)			3.95 (7.64)	
Albumin (g/L)				<0.001	0.877		0.800
Intervention groups	37.20 (3.37)	34.64 (4.387)	38.98 (5.22)			1.78 (5.60)	
Control groups	37.12 (4.68)	34.26 (5.19)	39.21 (3.68)			2.09 (5.93)	
Weight (kg)				<0.001	0.04		0.026
Intervention groups	65.64 (9.56)	65.57 (9.47)	63.75 (9.77)			−1.89 (2.14)	
Control groups	64.01 (10.92)	63.62 (10.77)	61.21 (10.93)			−2.80 (2.19)	
BMI				<0.001	0.029		0.014
Intervention groups	24.19 (3.05)	24.14 (3.02)	23.53 (3.12)			−0.65 (0.72)	
Control groups	23.38 (2.64)	23.19 (2.61)	22.36 (2.77)			−1.02 (0.84)	
Upper arm circumference (cm)				0.124	0.067		0.072
Intervention groups	27.35 (2.62)	27.35 (2.62)	27.03 (2.84)			−0.33 (0.88)	
Control groups	26.72 (2.61)	26.57 (2.70)	26.80 (2.85)			−0.07 (1.39)	
Handgrip strength (kg)				0.02	0.464		0.827
Intervention groups	28.65 (8.45)	28.56 (8.46)	28.06 (7.88)			−0.59 (2.47)	
Control groups	27.10 (7.69)	26.80 (7.59)	26.63 (7.12)			−0.46 (3.49)	
Calf circumference (cm)				<0.001	0.632		0.502
Intervention groups	34.65 (2.97)	34.61 (2.99)	34.19 (2.96)			−0.45 (0.28)	
Control groups	34.04 (3.33)	33.92 (3.29)	33.55 (3.27)			−0.49 (0.26)	
Body water (%)				0.597	0.691		0.47
Intervention groups	34.42 (5.46)	34.36 (5.45)	34.49 (5.97)			0.06 (3.00)	
Control groups	34.13 (5.81)	34.11 (5.81)	33.77 (5.70)			−0.36 (2.85)	
Muscle mass (kg)				0.004	0.356		0.838
Intervention groups	42.15 (9.75)	42.09 (9.70)	44.16 (7.69)			2.01 (6.28)	
Control groups	40.05 (9.24)	39.72 (9.22)	41.78 (7.20)			1.73 (7.33)	
Fat-free mass (kg)				0.918	0.274		0.432
Intervention groups	46.41 (7.33)	46.43 (7.30)	46.56 (8.08)			0.15 (4.17)	
Control groups	46.28 (7.89)	46.26 (7.91)	45.81 (7.69)			−0.47 (3.80)	
Skeletal muscle mass (kg)				0.052	0.69		0.69
Intervention groups	24.77 (4.83)	23.87 (4.83)	25.34 (4.88)			0.57 (2.39)	
Control groups	24.46 (4.55)	23.56 (4.55)	24.84 (4.56)			0.38 (2.51)	
Body fat mass (kg)				0.014	0.528		0.667
Intervention groups	18.54 (6.79)	18.54 (6.70)	17.63 (6.12)			−0.91 (4.18)	
Control groups	16.55 (5.67)	16.51 (5.63)	15.29 (5.03)			−1.26 (4.03)	
Body fat percentage (%)				0.01	0.563		0.885
Intervention groups	28.70 (8.35)	28.70 (8.07)	27.05 (8.16)			−1.64 (6.23)	
Control groups	27.32 (6.65)	27.28 (6.60)	25.10 (5.94)			−1.43 (7.88)	
Appendicular skeletal muscle mass (kg)				<0.001	<0.001		0.905
Intervention groups	19.44 (3.92)	18.44 (3.92)	20.83 (3.93)			3.67 (6.40)	
Control groups	19.13 (4.13)	17.77 (4.10)	18.62 (3.97)			3.49 (8.76)	
Appendicular skeletal muscle index (kg/m^2^)				<0.001	<0.001		<0.001
Intervention groups	6.92 (1.40)	6.72 (1.02)	7.41 (1.41)			0.49 (0.69)	
Control groups	7.04 (1.17)	6.50 (1.19)	6.83 (0.96)			−0.21 (1.20)	
Fat-free mass index (kg/m^2^)				0.723	0.307		0.52
Intervention groups	17.09 (1.76)	17.10 (1.74)	17.07 (1.92)			0.49 (0.69)	
Control groups	16.93 (1.77)	16.92 (1.78)	16.72 (1.76)			−0.21 (1.20)	
Fried frailty score				0.313	0.024		0.018
Intervention groups	1.29 (1.17)	1.27 (1.14)	0.98 (0.77)			−0.32 (0.99)	
Control groups	1.17 (0.99)	1.19 (1.03)	1.29 (1.12)			0.12 (0.96)	
NRS 2002 total score				<0.001	0.800		0.569
Intervention groups	3.23 (0.82)	3.19 (0.80)	2.07 (0.92)			−1.16 (0.84)	
Control groups	3.33 (1.02)	3.31 (1.00)	2.28 (1.17)			−1.05 (1.13)	

P_1_ denotes the group effect, indicating whether significant differences exist in overall temporal trends between the intervention and control groups. P_2_ represents the interaction effect, evaluating whether the magnitude of change varies significantly over time or across other factors. P_3_ assesses the absolute between-group difference in postoperative changes, comparing the absolute delta from baseline to 30-day post-surgery values between groups.

Postoperative changes from baseline to 30 days post-discharge demonstrated greater improvements in the intervention group for weight (*p* = 0.026), BMI (*p* = 0.014), ASMI (*p* < 0.001), and frailty scores (*p* = 0.018). Notably, while both groups experienced postoperative weight loss, the intervention group maintained better weight stability (−1.89 ± 2.14 kg vs. −2.80 ± 2.19 kg; *p* = 0.026) and BMI (−0.65 ± 0.72 vs. −1.02 ± 0.84 kg/m^2^; *p* = 0.014), alongside increased muscle mass (ASMI: 0.49 ± 0.69 vs. −0.21 ± 1.20; *p* < 0.001). Fat mass reduction showed a non-significant trend toward preservation (−0.91 ± 4.18 vs. −1.26 ± 4.03; *p* = 0.667).

## 4 Discussion

This RCT evaluated the efficacy of MDT-based perioperative nutritional management in older surgical patients. The intervention group showed a significantly lower 30-day postoperative complication rate and a non-significant reduction in in-hospital complications. Post-discharge complications were comparable. The intervention group demonstrated a 6.2-point lower CCI score and a 3.67-day shorter median postoperative hospital stay. Key outcomes included reduced complications and accelerated recovery, with statistical significance in hospital stay duration.

The intervention group demonstrated significant benefits in functional recovery (mobility, pain relief) and reduced postoperative complications [16.1% reduction vs. 12.6% in Molenaar et al. (5)], likely due to longer intervention duration (preoperative to 30-day post-discharge vs. 4 weeks). This MDT-based model improved outcomes in high-risk older adults with comorbidities, showing metabolic advantages from prolonged intervention. Hospital costs were 9.7% lower, suggesting potential economic benefits. While we observed a non-significant reduction in hospitalization costs, future studies incorporating formal cost-effectiveness analyses are warranted to validate the economic impact of MDT-based nutritional management. Comprehensive assessments revealed regulatory effects on nutritional/metabolic status through muscle mass, body composition, and frailty evaluations, collectively supporting the model’s value in optimizing perioperative care for older surgical patients.

This study advances perioperative rehabilitation methodology through innovative intervention design, population targeting, and analytical rigor. Unlike prior single-component approaches [e.g., nutritional support (14) or exercise (15)], our MDT-based model integrates nutrition, exercise, and psychological interventions synergistically. While Gao et al. (10) showed preoperative PN reduced infections by 9.7% without exercise, our multimodal strategy decreased complications and improved muscle mass. What we would like to emphasize is that although this study did not separate the effects of each intervention, this was done to reflect a comprehensive nursing model that is commonly applied in clinical settings. The intervention group demonstrated a 6.2-point lower CCI score than controls, indicating meaningful complication reduction exceeding Francesco et al.’s findings (16), though statistical significance was not achieved. Notably, Bousquet-Dion et al. (17) reported 23% anxiety improvement in colorectal patients without psychological interventions, whereas our audio-visual counseling achieved 26.8% improvement in post-discharge anxiety/depression. Unlike previous studies focusing on general adults or specific procedures (e.g., colorectal surgery), this trial exclusively enrolled high-risk older adults (≥65y) undergoing major abdominal surgery—a cohort with 2-3× higher complication risks due to sarcopenia and frailty (18). Despite elevated baseline risks, our intervention reduced complications more effectively than Carli et al.’s preoperative rehabilitation (16), which showed limited effects in general surgical populations. While acknowledging the methodological limitation of integrated intervention components (preventing isolation of individual effects), this design mirrors real-world multimodal clinical practice. Observed benefits likely stem from synergistic interactions between nutritional, exercise, and psychological elements in the MDT-based model.

Perioperative comprehensive nutritional management, a precision nutrition cornerstone, shows superior clinical benefits over conventional methods, especially in nutritionally at-risk patients. Xu et al. (19)‘s single-center RCT in pancreaticoduodenectomy patients demonstrated significant reductions in overall/infectious complications and intra-abdominal infections with multimodal management. Comprehensive therapy throughout the perioperative period enhances muscle protein synthesis (16), correlating with greater postoperative limb skeletal muscle mass retention in the intervention group. This muscle preservation likely reduces adverse outcomes in older adults (20). Notably, the intervention group experienced smaller postoperative weight loss (−1.89 kg vs. −2.80 kg, *p* = 0.026) and slower BMI decline (−0.65 vs. −1.02, *p* = 0.014), suggesting comprehensive nutrition buffers surgical stress-induced energy hyperconsumption. These findings highlight the model’s potential to mitigate metabolic disturbances and improve clinical trajectories in high-risk surgical populations. This study, through nutritional intervention within the MDT model, significantly improved the post-operative recovery of elderly patients. Particularly, it showed excellent results in reducing weight loss and maintaining muscle mass.

Repeated-measures ANOVA revealed time-dependent effects of the MDT model: ASMM showed progressive improvement post-discharge (*p* < 0.001), with widening intergroup differences over time. This pattern aligns with the cumulative effects of resistance training, which stimulates muscle satellite cell proliferation through mechanical loading, thereby counteracting postoperative muscle atrophy. It is worth noting that the nutritional support during the intervention process provides the necessary material basis for muscle remodeling. The smaller grip strength reduction in the intervention group (*p* = 0.089) is particularly noteworthy, as handgrip strength serves as a key predictor of postoperative functional independence in sarcopenic patients (21). Although the absolute change in grip strength and the changes in grip strength between the two groups did not have statistically significant trends over time, there was a significant difference in the overall trend of grip strength between the intervention group and the control group. These findings should be further explored in larger-scale trials. The smaller reductions in weight and BMI observed in the intervention group (*p* = 0.014), coupled with the time-dependent nature of these differences (*p* = 0.029), suggest that enhanced preoperative nutritional reserves may have buffered against postoperative metabolic stress. This mechanism, challenging to replicate with traditional single-component interventions, underscores the metabolic integration advantage of MDT-based multidimensional rehabilitation.

Several limitations require attention. First, the 30-day follow-up and 120-patient sample limit long-term evaluation; future studies need longer follow-up (6–12 months). Second, while baseline groups were comparable, individual disease progression and personalized treatment decisions may introduce heterogeneity. Third, as a single-center study with predominantly Han Chinese participants, findings may lack generalizability; multi-center international trials are warranted. The small sample size of this study may require cautious interpretation of some results that did not reach statistical significance (such as CCI scores and anxiety). It is recommended that larger-scale studies be conducted in the future to verify these findings. Fourth, home-based intervention adherence varied despite digital monitoring; AI-driven real-time adjustments could improve compliance. Although digital monitoring was used to track patients’ compliance, we did not have detailed records of their compliance with the exercise and nutrition plans. Future research should employ more precise monitoring methods (such as real-time data capture) to better track compliance. Furthermore, the inclusion of various common abdominal surgeries (such as gastrointestinal surgeries and liver-biliary surgeries) may lead to differences in complication risks and recovery patterns. Although the baseline characteristics were balanced, due to the limitation of sample size, we did not conduct subgroup analyses. Future studies with larger sample sizes should explore the specific outcomes of the surgeries. Finally, lack of direct muscle protein synthesis or inflammatory biomarker measurements limits mechanistic insights; future trials should integrate multi-omics analyses.

## 5 Conclusion

This RCT confirms the clinical efficacy of MDT-based perioperative nutritional management in older surgical patients through an integrated care continuum (preoperative, postoperative, post-discharge) with exercise and psychological support. Future priorities include long-term outcome evaluation, mechanistic exploration via advanced omics, and AI-driven personalized intervention models to advance geriatric perioperative care paradigms.

## Data Availability

The original contributions presented in the study are included in the article/supplementary material, further inquiries can be directed to the corresponding authors.

## Footnotes

1
http://www.assessurgery.com
